# Prediction of corneal graft rejection using central endothelium/Descemet’s membrane complex thickness in high-risk corneal transplants

**DOI:** 10.1038/s41598-021-93892-4

**Published:** 2021-07-15

**Authors:** Taher Eleiwa, Amr Elsawy, Eyup Ozcan, Collin Chase, William Feuer, Sonia H. Yoo, Victor L. Perez, Mohamed F. Abou Shousha

**Affiliations:** 1 Bascom Palmer Eye Institute, Miller School of Medicine, University of Miami, 900 NW 17 Street, Miami, FL 33136 USA; 2 Department of Ophthalmology, Faculty of Medicine, Benha University, Benha, Egypt; 3 Electrical and Computer Engineering, University of Miami, Miami, FL USA; 4 Biomedical Engineering, University of Miami, Miami, FL USA; 5 Morsani College of Medicine, University of South Florida, Tampa, FL USA; 6 Duke Eye Center, Duke University School of Medicine, Durham, NC USA

**Keywords:** Corneal diseases, Three-dimensional imaging

## Abstract

To determine whether measurements of Endothelium/Descemet complex thickness (En/DMT) are of predictive value for corneal graft rejection after high-risk corneal transplantation, we conducted this prospective, single-center, observational case series including sixty eyes (60 patients) at high risk for corneal graft rejection (GR) because of previous immunologic graft failure or having at least two quadrants of stromal vascularization. Patients underwent corneal transplant. At 1st, 3rd, 6th, 9th, and 12th postoperative month, HD-OCT imaging of the cornea was performed, and the corneal status was determined clinically at each visit by a masked cornea specialist. Custom-built segmentation tomography algorithm was used to measure the central En/DMT. Relationships between baseline factors and En/DMT were explored. Time dependent covariate Cox survival regression was used to assess the effect of post-operative En/DMT changes during follow up. A longitudinal repeated measures model was used to assess the relationship between En/DMT and graft status. Outcome measures included graft rejection, central Endothelium/Descemet’s complex thickness, and central corneal thickness (CCT). In patients with GR (35%), the central En/DMT increased significantly 5.3 months (95% CI: 2, 11) prior to the clinical diagnosis of GR, while it remained stable in patients without GR. During the 1-year follow up, the rejected grafts have higher mean pre-rejection En/DMTs (*p* = 0.01), compared to CCTs (*p* = 0.7). For En/DMT ≥ 18 µm cut-off (at any pre-rejection visit), the Cox proportional hazard ratio was 6.89 (95% CI: 2.03, 23.4; *p* = 0.002), and it increased to 9.91 (95% CI: 3.32, 29.6; *p* < 0.001) with a ≥ 19 µm cut-off. In high-risk corneal transplants, the increase in En/DMT allowed predicting rejection prior to the clinical diagnosis.

## Introduction

Corneal graft rejection is the most common cause of graft failure in the intermediate and late postoperative period. Up to 30% of the yearly 60,000 corneal transplant patients experience at least one episode of rejection^1,2^. Despite the use of immunosuppressants, graft failure rate secondary to a rejection varies from 5% in low-risk penetrating corneal transplant (PK) after five years to 35% in high-risk PK at three years^1^. These failures inflict a heavy burden on the patients’ productivity and the health care system^1^. Therefore, early diagnosis of graft rejection and aggressive therapy with corticosteroids are crucial to long-term graft survival for high-risk corneal transplant recipients.

Early diagnosis of graft rejection is necessary to initiate the treatment before irreversible endothelial cell damage to improve graft survivals^3^. Increased central corneal thickness may offer an early warning of rejection; however, central corneal thickness alone is not a reliable index of graft immunological status^4,5^. Using specular microscopy, a significant decline in the endothelial cell density in severe graft rejection was reported, while mild rejection episodes were not associated with a loss in cell density surpassing the expected^6^. Using contact confocal microscopy, central immune cell densities have been reported to increase prior to graft rejection via manual counting^7^. Likewise, aqueous humor cytokine levels have been reported to increase during rejection of PK^8^. In addition, retrospective analysis of specular microscopy and Scheimpflug imaging has been shown to improve detection of allograft rejection following DMEK^9^. Using anterior segment OCT (AS-OCT), endothelial/Descemet membrane complex thickness (En/DMT) has been reported to accurately assess the immunological status of the corneal graft as compared to both endothelial cell density, and central corneal thickness^10,11^.

Recently, we developed and validated a segmental tomography algorithm to generate 3D thickness maps of different corneal layers using AS-OCT corneal scans to enhance the diagnosis of different corneal diseases^12–15^. Using this tool, the three-dimensional thickness of the endothelial/Descemet membrane complex has shown excellent sensitivity and specificity in diagnosing corneal graft rejection that strongly correlated with the rejection severity^13^. Hereafter, we hypothesized that En/DMT could potentially reveal early changes that herald corneal graft rejection. In this study, we report on the use of segmental tomography algorithm, and examine the relationship between the central three-dimensional (3D) En/DMT and graft rejection in this population at high risk for rejection.

## Materials and methods

### Study design and participants

This study was approved by the University of Miami Institutional Review Board. All participants provided written informed consent before enrollment. The study design followed the tenets of the Declaration of Helsinki for biomedical research.

Sixty eyes of 60 patients were prospectively and consecutively recruited from 2016 to August 2019 at Bascom Palmer Eye Institute, University of Miami. For inclusion in the study, eyes were required to have a high-risk feature for graft rejection as described elsewhere^2^. Corneas eligible for transplantation were from donors aged 10 to 70 years with a preoperative endothelial cell density (ECD) of at least 2500 cells/mm^2^ determined by the eye bank. Surgical technique and perioperative care were provided according to each surgeon’s customary routine. Every patient was required to complete regularly scheduled postoperative ophthalmic examinations at 1 week, 2 weeks, and 1, 3, 6, 9, and 12 months after surgery. Patients were instructed to contact the surgeon immediately if they had a dropped vision, redness, photophobia, or pain. At each visit, the slit-lamp examination was performed on each eye by a cornea specialist, masked to the OCT data, to assign the examined cornea into either a healthy, rejection or failed category. Corneal graft rejections were considered as the primary endpoint in this study. Endothelial graft rejection was diagnosed by detecting new keratic precipitates (KPs) or a Khodadoust line in the presence of anterior chamber cells and new persistent corneal graft edema in a graft that was previously clear^2^. Failed graft was considered if there was an irreversible loss of central graft clarity for a minimum of 3 consecutive months after endothelial rejection. Exclusion criteria included epithelial or stromal rejections and microbial infection after corneal transplantation.

### Image acquisition and analysis

Anterior segment HD-OCT (Envisu R2210, Bioptigen, Buffalo Grove, IL, USA) with 6 mm radial cuts centered on the corneal vertex was performed at 1st, 3rd, 6th, 9th, and 12th month after surgery. The characteristics of this device were described in our previous work^13,16^. To confirm optimal centration, each candidate was asked to fix at a target to visualize a saturation artifact in all images. The fixation target was customized in decentered grafts and post-PK eyes with high irregular astigmatism to maintain the geometric centration of the scan upon the graft. A custom-built segmental tomography algorithm was used to automatically generate the 3D thickness of the En/DM in the central 2 mm region of the corneal transplant. The automatic segmentation was checked by 2 experts, masked to the clinical diagnosis, with manual editing of segmented images if needed. We have reported excellent reliability of our image processing techniques for the total corneal thickness and En/DMT measurements in healthy corneas, Fuchs’ endothelial cell dystrophy, and corneal graft rejection^12–14,16^. In Brief, Random Sample Consensus (RANSAC)^17^ method with a polynomial model is used to estimate the borders of corneal layers. Then, reconstruction of the corneal layers is done via bi-cubic interpolation^18^. Lastly, 3D ray-tracing algorithm is used to generate the mean 3D thickness values of the central 2-mm region of the corneal transplant^19,20^.

### Diagnostic indices

Central 3D-En/DM complex and central corneal thickness were the diagnostic indices generated from the segmented HD-OCT images. We previously reported the 2 interfaces of the En/DM as the two most posterior hyperreflective bands of the cornea on HD-OCT images^13,21^.

### Statistical analysis

Statistical analyses were performed using SPSS software version 22.0 (SPSS, Chicago, IL, USA) to calculate descriptive statistics for all eyes. The obtained CCT measurements were verified to have a normal distribution by assessment of histograms and a Shapiro–Wilk’s test of normality, while En/DMT was not. Therefore, mean ± standard deviations (SD) were used to characterize the distribution of the corneal thickness values, while the median values were used to characterize En/DMT.

Multivariate analyses were used to explore the relationships between baseline (donor, recipient, and operative) factors and En/DMT. Longitudinal repeated measures models were used to evaluate En/DMT change throughout the follow-up. The final multivariable model was generated through a stepwise selection of covariates at a significance level of 0.05.

We used time-dependent covariate Cox survival regression to assess the effect of post-operative En/DMT changes on the risk of graft rejection. For this purpose, a variety of cut points for En/DMT thickness, measured at the 1, 3, and 6 months follow-up visits were specified prior to data analysis. These were included in a series of time-dependent proportional hazards models to estimate the increased risk of graft rejection with attainment of each En/DMT thickness cut point, postoperatively. The time-dependent Cox analyses estimate the increase in risk associated with each En/DMT cutpoint, regardless of follow up time. They are expressed as ratios of rejection risk in eyes with En/DMT thicker than the cutpoint to eyes with En/DMT thinner than the cutpoint. These risk ratios apply whenever the En/DMT thickness is observed to be greater than the cutpoint and are not specific to any particular post-surgical follow up time.

## Results

The mean (± SD) age of the 60 patients included in the analysis was 59 ± 3 years, and 35 (58%) were males. At the beginning of the study, these participants underwent corneal transplantation (43 PK, 15 DSAEK, 2 DSAEK under PK) for the following indications: previous graft failure (35%), therapeutic (23%), pseudophakic bullous keratopathy (20%), herpetic eye disease (12%), vascularized leucoma-adherent after repaired corneal rupture (8%), and Stevens-Johnson Syndrome (2%). Table 1 summarizes the baseline recipient, donor and operative characteristics in the healthy grafts and the rejection grafts.

**Table 1:** Baseline recipient, donor and operative characteristics in the healthy grafts and the rejection grafts.

	Rejection group	Healthy group	*P* value
Eyes/patients (n)	21/21	39/39	–
Age (years), mean ± SD (range)	53 ± 4.62 (12–83)	63 ± 3.23 (16–94)	0.07*
**Gender**			
Male	9 (26%)	26 (74%)	0.10^†^
Female	12 (48%)	13 (52%)	
**Race**			
Non-white (Hispanic)	18 (37%)	31 (63%)	1.00^†^
White (non-Hispanic)	3 (30%)	7 (70%)	
**Postoperative intraocular lens status**			
Phakic	8 (40%)	12 (60%)	0.58^†^
Pseudophakic	13 (32%)	27 (68%)	
**Preoperative diagnosis**			
Failed graft	5	16	0.64^‡‡^
Therapeutic	6	8	
Pseudophakic Bullous Keratopathy	4	8	
Herpetic eye disease	3	4	
Stevens Johnson syndrome	1	0	
Post-traumatic vascularized Leucoma adherent	2	3	
**Number of vascularized quadrants**			
1 quadrant	0	0	0.010^‡^
2 quadrants	8 (24%)	25 (76%)	
3 quadrants	9 (39%)	14 (51%)	
4 quadrants	4 (100%)	0	
**Number of previous grafts**			
None	16 (41%)	23 (59%)	0.28^‡^
1 graft	3 (19%)	13 (81%)	
2 grafts	2 (40%)	3 (60%)	
Follow-up time (months), mean ± SD (range)	10 ± 0.64 (6–15)	11 ± 0.33 (1–12)	0.3
**Previous glaucoma surgery**			
None	11 (32%)	23 (68%)	0.79^†^
Yes	10 (38%)	16 (62%)	
**Transplant type**			
PK	16 (37%)	27 (63%)	0.69^‡^
DSAEK	4 (27%)	11 (73%)	
DSAEK under PK	1 (50%)	1 (50%)	
**Corneal button size**			
< 8 mm	11 (38%)	18 (62%)	0.077^‡^
= 8 mm	1 (8%)	11 (91%)	
> 8 mm	9 (47%)	10 (52%)	
**Postoperative IOP (1st visit)**			
≤ 25 mmHg	21 (37%)	35 (63%)	0.29^†^
> 25	0	4 (100%)	
**Donor age (years), mean ± SD** **(range)**			
	45 ± 3 (19–68)	46 ± 3 (18–69)	0.91*
**Donor gender**			
Male	17 (41%)	24 (59%)	0.15^†^
Female	4 (21%)	15 (79%)	
**Donor ethnicity**			
Non-white (Hispanic)	11 (42%)	15 (58%)	0.41^†^
White (non-hispanic)	10 (29%)	24 (71%)	
Baseline preoperative Endothelial Cell Density	3072 ± 74	2959 ± 53	0.22*

*Two sample t-test.

^†^Fisher exact test.

^‡^Chi-squared test.

^‡‡^Exact permutation Chi-square.

**Figure 1:**
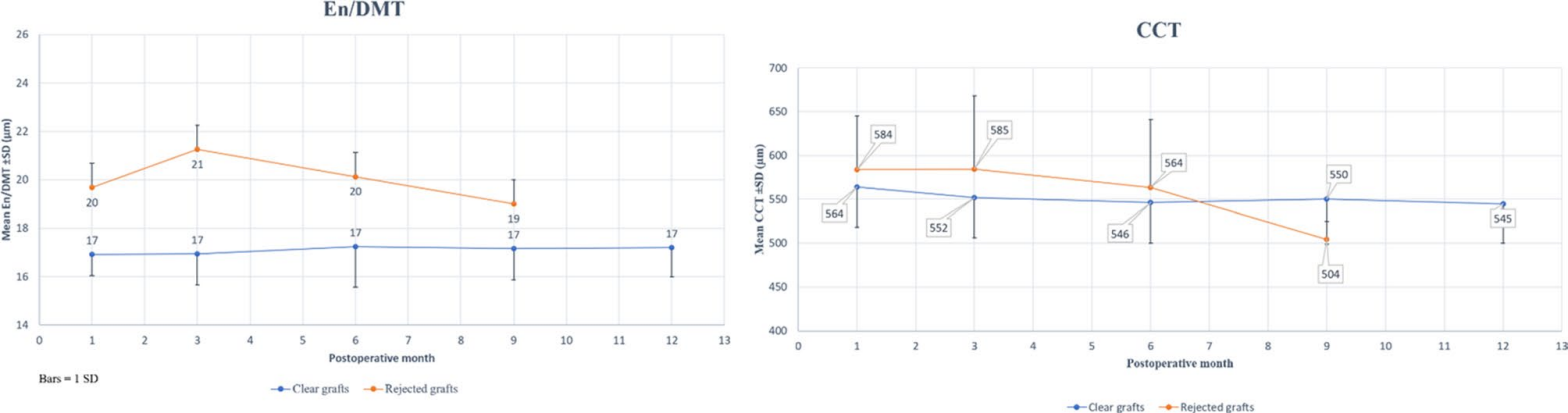
linear plots of the longitudinal repeated measures showing a significant increase (*p* = 0.01) in the mean central Endothelium-Descemet’s complex thickness (En/DMTs, left plot) in the corneal transplants, versus the insignificant changes (*p* = 0.7) of the central corneal thickness (CCT, right plot) through the visit prior to rejection during the 1st year postoperatively.

All HD-OCT images were segmented successfully, and no manual editing was required by both observers. Regarding the dynamic changes in En/DMT and central corneal thickness (CCT), Fig. 1 plots the longitudinal repeated measures of the mean En/DMTs and CCTs by the follow-up month for the grafts that remained clear but plots the rejected graft En/DMTs and CCTs only through the visit prior to rejection. Table 2 summarizes the number of analyzed cases at each time point. There is no rejected 12-month En/DMT or CCT means because the last pre-rejection values were measured at 9 months. The rejected grafts had a progressively higher mean pre-rejection En/DMTs (*p* = 0.01), as compared to the healthy grafts while the mean pre-rejection CCTs showed no significant changes (*p* = 0.7). It is shown that En/DMT was predictive of graft rejection with larger En/DMT values among the 21(35%) cases whose graft subsequently rejected compared with 39 non-rejection cases.

**Table 2:** Number of analyzed cases at each post-operative month (POM).

	POM#1	POM#3	POM#6	POM#9	POM#12
Clear corneal grafts	55	48	42	39	39
Corneal grafts in the visit prior to rejection	5	7	6	3	0

Time-dependent covariate Cox survival regression was used to assess the effect of post-operative En/DMT changes during follow-up. We explored the increased risk of graft rejection after En/DMT thickening with a variety of cut-points (Table 3). Using time-dependent covariate Cox proportional hazards regression to assess the risk of rejection if an eye has an En/DMT ≥ 18 µm (at any previous rejection visit). For this cut-off, the risk ratio was 6.89 (95% CI: 2.03, 23.4; *p* = 0.002). Increasing En/DMT cut-off to 19 µm, the risk increases to 9.91 (95% CI: 3.32, 29.6). This data-driven analysis determined that once En/DMT thickened to 19 µm or greater the risk of graft rejection increased by a factor of almost 10. Of the 21 grafts that were rejected, 17 (81%) had a postoperative progressively increasing En/DMT ≥ 19 µm prior to rejection, while of 39 grafts remaining clear only 7 (18%) had a postoperative stable En/DMT ≥ 19 µm as compared to their respective baseline measurements. For cases where En/DMT thickened to 19 µm, graft rejection did not necessarily follow immediately, the range of intervals was 2 to 11 months (mean = 5.3). The time dependent Cox analysis produces estimates for increased risk that are not specific to follow up time; therefore, the observation of an En/DMT thicker than 18um or 19um is concerning whenever it occurs during follow up.

**Table 3:** Time dependent covariate Cox survival regression to assess the effect of post-operative central endothelium/Descemet’s membrane complex thickness (En/DMT) changes during follow up. We explored the increased risk of graft rejection after En/DMT thickening with a variety of cut-points.

Central En/DMT greater than or equal to (µm)	Hazard ratio (95% CI)	*P* value
17	3.35 (0.8, 14.4)	0.057
18	6.89 (2.0, 13.4)	0.002
19	9.91 (3.3, 29.6)	< 0.001
20	6.51 (2.7, 15.9)	< 0.001
21	5.4 (2.2, 13.1)	< 0.001

Qualitatively, Fig. 1 demonstrated the relative thickening of the En/DM and the appearance of focal excrescences in the visit (Fig. 1B) prior to the clinical diagnosis of rejection via the masked cornea specialist (Fig. 1C), as compared to the healthy En/DM of the same eye in the visit before (Fig. 1A). In the 18% of the healthy grafts with En/DMT ≥ 19 µm, no focal irregularities were noted in the central En/DM till the last follow-up visit.

Running the time-dependent covariate analysis with the En/DMT cut point set to 19 µm and allowing forward stepwise inclusion of all the continuous variables as well as the indication for and type of the corneal transplant was done. Of these, the only variable that entered the model was age (*p* = 0.026), which did not affect the significance of En/DMT (still *p* < 0.001). In this model, the risk of rejection increases by a factor of 10.9 times (95% CI: 3.6, 32.8) after En/DMT reaches a thickness of 19 µm. The effect of age was that risk of rejection decreases by a factor of 0.19 (95% CI: 0.03, 0.32), about 20% with a 10-year increase in age. The only other variable which came close to statistical significance was transplant type (*p* = 0.079) but it did not enter the model because *p* > 0.05.

## Discussion

In high-risk corneal transplantation, the rejection rates can exceed 70% despite local and systemic immune suppression^22^. Acknowledging the high failure rate secondary to rejection in these high-risk corneas, it is critical that we develop measures that help diagnose graft rejection early to precociously start therapy before permanent endothelial cell damage. It has been established in the literature that thickening of basement membranes of allografts is a sign of rejection in solid-organ transplantation. Likewise, thickening of the Descemet membrane has been reported to reflect the immunological status of corneal grafts^13,21,23^. Recently, we reported the feasibility of implementing corneal segmental tomography algorithm in generating 3D thickness maps of corneal layers^12,14^. Those studies have shown the great potential of using this technology to describe the in vivo microstructure of the corneal En/DM and its promising utility in the diagnosis of endothelial pathologies such as corneal graft rejection and Fuchs’ endothelial dystrophy. Using this tool, En/DM was found to have excellent sensitivity and specificity in diagnosing active corneal graft rejection that strongly correlated with the rejection severity, compared to CT and ECC^21,24^.

To date, variable tools have been reported to predict corneal graft rejection. In Cornea Donor Study, increasing CT was reported as an independent predictor of graft survival at 6 months to 5 years of follow-up after PK. Also, the Collaborative Corneal Transplantation Studies reported that increased CT at 1, 3, and 6 months post-operative were predictive of graft failure in high-risk penetrating corneal transplant eyes followed for 3 years^25^. However, both groups were restricted to PK and no endothelial replacement graft was included. In addition, single CT could not be used solely to diagnose rejection owing to the wide variations reported of CT in healthy corneas. In the same context, sequential differential 360-degree Scheimpflug imaging has been recently reported, retrospectively, after DMEK to predict a rejection episode 8 ± 3 months before clinically manifested^26^. Using confocal microscopy, subjective manual counting of activated keratocytes was reported to predict graft rejection 2 months prior to the clinical diagnosis^22^. Furthermore, invasive aqueous humor cytokine analysis was found to predict immunological reaction fo’’llowing PK^8^.

**Figure 2:**
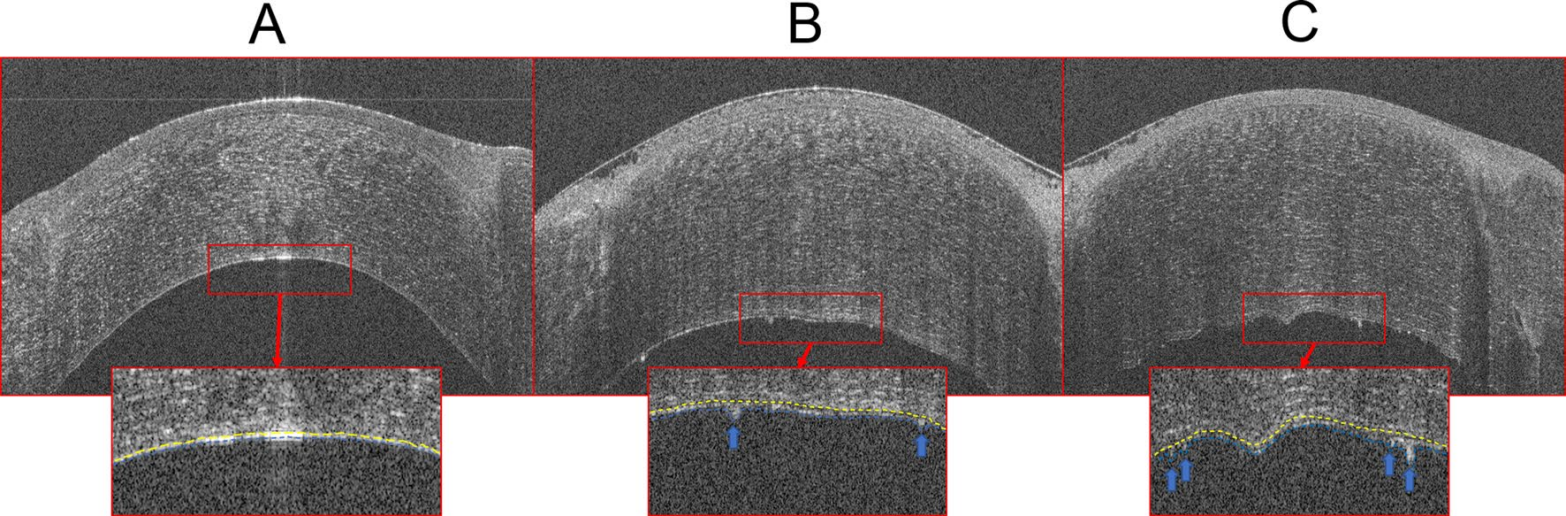
Chronological high-definition optical coherence tomography (HD-OCT) images of a full-thickness corneal transplant of one eye of the same patient (upper raw) with magnified preset images of the corresponding central endothelium (yellow-dashed line)/Descemet’s membrane (blue-dashed line) complex (En/DM, lower raw) showing: (**A)** and (**B**) HD-OCT image of clinically healthy corneal graft at the 6th and 9th postoperative months, respectively, with the En/DM visualized as a band formed by 2 smooth hyperreflective lines with a translucent space in between in (**A**), and as a thickened band bounded by 2 hyperreflective lines with 2 posterior nodular excrescences (arrows) in (**B**). (**C)** HD-OCT image of clinically diagnosed graft rejection at the 1-year visit showing the En/DM as a thick wavy band bounded by 2 hyperreflective lines with 4 posterior nodular excrescences (arrows).

In the current study, we reported the predictive role of the central 3D thickness of En/DM in corneal graft rejection. We have included high-risk corneal transplants due to the higher rates of rejection. PK, DSAEK and DSAEK under PK were included in our study. We reported a 35% one-year rate of rejection in our cohort, within the range of those reported in other series of patients who underwent high-risk corneal transplant^1,27^. Using segmentation tomography algorithm, a progressive thickening of the En/DMT in the central 2-mm region of the corneal transplant was found to predict a rejection in the first 6 months in PK and DSAEK, as compared to CCT (Fig. 2). Previously, we reported that En/DMT was not affected by the type of the corneal transplant^13^. In healthy grafts, a stable En/DMT was found throughout the follow-up period of 1 year, compared to CCT which was reported to decline due to the recovery of the donor endothelium after the initial insult of surgery^28^. It is noteworthy to mention that in the clinically healthy grafts with a thicker En/DMT compared to their respective baseline measurements, the En/DM was smooth and remained stable during the subsequent follow-up visits till the end of the study (postoperative one-year). This thickening could be explained by a resolved subtle rejection episode that might have occurred in-between the follow-up visits and was not evident during the clinical examination. In our series, the risk of rejection decreases about 20% with a 10-year increase in age, in agreement with Vabres et al.^29^ and Alexandre et al.^30^ who reported that the rejection rate decreases with aging. Future studies are required to allow better utilization of En/DMT as a way of evaluating prophylaxis or treatment measures for graft rejection. For example, if low-grade rejection or inflammation exists as a cause of graft failure, more frequent steroid treatment might be evaluated based on En/DMT measurements.

There are a few limitations in our study. First, our results stemmed from a limited number of patients, and a larger study is required to replicate our results with longer follow-up time. Second, owing to the high-risk nature of the recipient beds, our study was limited to the central En/DMT due to the poor signal to noise ratio in the periphery of the OCT images that hinder the ability to delineate the corneal En/DM consistently in all the frames. Hypothetically, an ongoing rejection in the periphery could be missed. Despite this limitation, central En/DMT was found to increase at least 2 months prior to clinically detected rejection. Third, our cohort was restricted to eyes at high-risk of graft rejection. Eyes at low to mid risk of graft rejection following corneal transplantation should be included in future studies with longer follow-up duration. Lastly, the influence of possible confounding factors on the results of our series such as differences in surgical techniques could not be assessed.

In conclusion, in our series, a major advantage for En/DMT compared to CT is that a one-time measurement could predict graft rejection in high-risk corneal transplants during the first year postoperatively. The progressive thickening and the appearance of nodular excrescences of the En/DM compared to the previous visit should raise a red flag of an upcoming or existing rejection. Also, as an objective parameter of graft immunological status, we believe that En/DMT would be appropriately included in any future studies examining graft survival after a high-risk corneal transplantation.

### Ethics approval and consent to participate

This study was approved by the University of Miami Institutional Review Board.

## Data Availability

The datasets used and/or analyzed during the current study available from the corresponding author on reasonable request.

## Abbreviations

En/DM: Endothelium-Descemet’s complex
En/DMT: Endothelium-Descemet’s complex thickness
TCT: Total corneal thickness
CCT: Central corneal thickness
HD-OCT: High-definition optical coherence tomography
AS-OCT: Anterior segment optical coherence tomography
ROC: Receiver operating characteristics
AUC: Area under curve
OCV: Optimal cut-off value
PK: Penetrating corneal transplant
DSAEK: Descemet stripping automated endothelial corneal transplant
SD: Standard deviation
ANOVA: Analysis of variance
2D: Two-dimensional
3D: Three-dimensional
Epi: Epithelium
KPs: Keratic precipitates
